# Author Correction: A monofluoride ether-based electrolyte solution for fast-charging and low-temperature non-aqueous lithium metal batteries

**DOI:** 10.1038/s41467-023-40318-6

**Published:** 2023-07-27

**Authors:** Guangzhao Zhang, Jian Chang, Liguang Wang, Jiawei Li, Chaoyang Wang, Ruo Wang, Guoli Shi, Kai Yu, Wei Huang, Honghe Zheng, Tianpin Wu, Yonghong Deng, Jun Lu

**Affiliations:** 1 Department of Materials Science & Engineering, School of Innovation and Entrepreneurship, Southern University of Science and Technology, Southern University of Science and Technology of China, Shenzhen, 518055 China; 2 College of Chemical and Biological Engineering, Zhejiang University, Hangzhou, 310027 China; 3 School of Materials Science and Engineering, China University of Petroleum (East China) Qingdao, Qingdao, 266580 China; 4 Research Institute of Materials Science, South China University of Technology, Guangzhou, 510640 China; 5 National Center for Applied Mathematics Shenzhen (NCAMS, Digital Economy Research Center-DeFin) and College of Business, Southern University of Science and Technology, Shenzhen, 518055 China; 6 College of Energy & Collaborative Innovation Center of Suzhou Nano Science and Technology, Soochow University, Suzhou, Jiangsu 215006 China

**Keywords:** Batteries, Materials science, Energy storage, Energy, Materials for energy and catalysis

Correction to: *Nature Communications* 10.1038/s41467-023-36793-6, published online 25 February 2023

Supplementary Figure 30 of this article contained an error. This error is now corrected with a new figure.

## Supplementary information

Updated Supplementary Information

