# Supplementary material for: A monofluoride ether-based electrolyte solution for fast-charging and low-temperature non-aqueous lithium metal batteries
Source: Nat Commun. 2023 Feb 25;14:1081. doi: 10.1038/s41467-023-36793-6 (PMC9968335; doi:10.1038/s41467-023-36793-6)
Supplement: Supplementary file 1 — Supplementary Information [file 41467_2023_36793_MOESM1_ESM.docx]

Supplementary Information

**A monofluoride ether-based electrolyte solution for fast-charging and low-temperature non-aqueous lithium metal batteries**

Guangzhao Zhang,^1,7^ Jian Chang,^1,7^ Liguang Wang,^2^ Jiawei Li,^3^ Chaoyang Wang,^4^ Ruo Wang,^1^ Guoli Shi,^1^ Kai Yu,^1^ Wei Huang,^5^ Honghe Zheng,^6^ Tianpin Wu,^2^* Yonghong Deng^1^* and Jun Lu^2^*

^1^Department of Materials Science & Engineering, School of Innovation and Entrepreneurship, Southern University of Science and Technology, Shenzhen 518055, China.

^2^College of Chemical and Biological Engineering, Zhejiang University, Hangzhou, 310027 China.

^3^School of Materials Science and Engineering, China University of Petroleum (East China) Qingdao 266580, China.

^4^Research Institute of Materials Science, South China University of Technology, Guangzhou 510640, China.

^5^National Center for Applied Mathematics Shenzhen (NCAMS, Digital Economy Research Center-DeFin) and College of Business, Southern University of Science and Technology, Shenzhen 518055, China.

^6^College of Energy & Collaborative Innovation Center of Suzhou Nano Science and Technology, Soochow University, Suzhou, Jiangsu 215006, China.

^7^These authors contributed equally: Guangzhao Zhang and Jian Chang

*Corresponding Author. yhdeng08@163.com (Y. Deng); junzoelu@zju.edu.cn (J. Lu) and tianpinwu@zju.edu.cn (T. Wu)


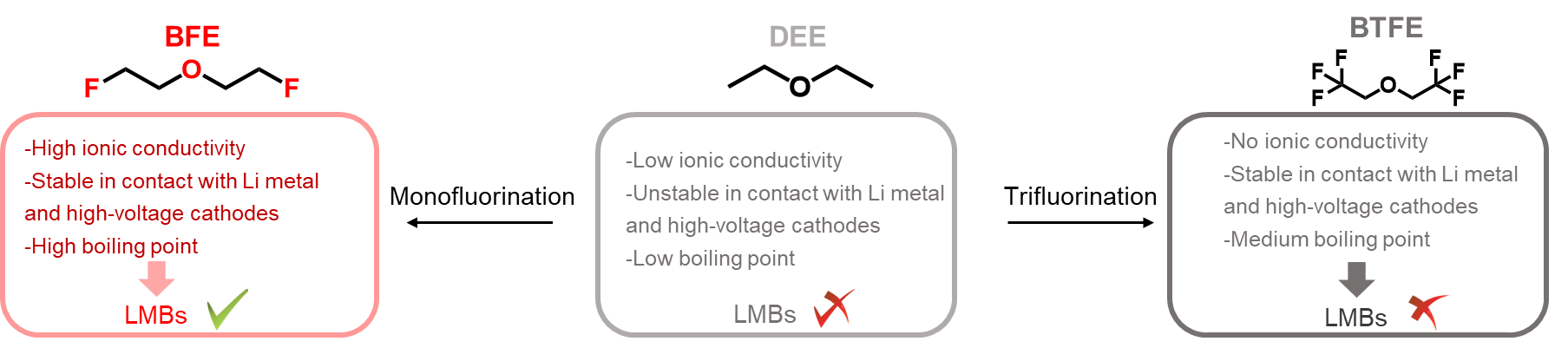


**Supplementary Figure 1.** Molecular design of BFE versus nonfluoro- (DEE) and trifluoro- (BTFE) substituted ethers.


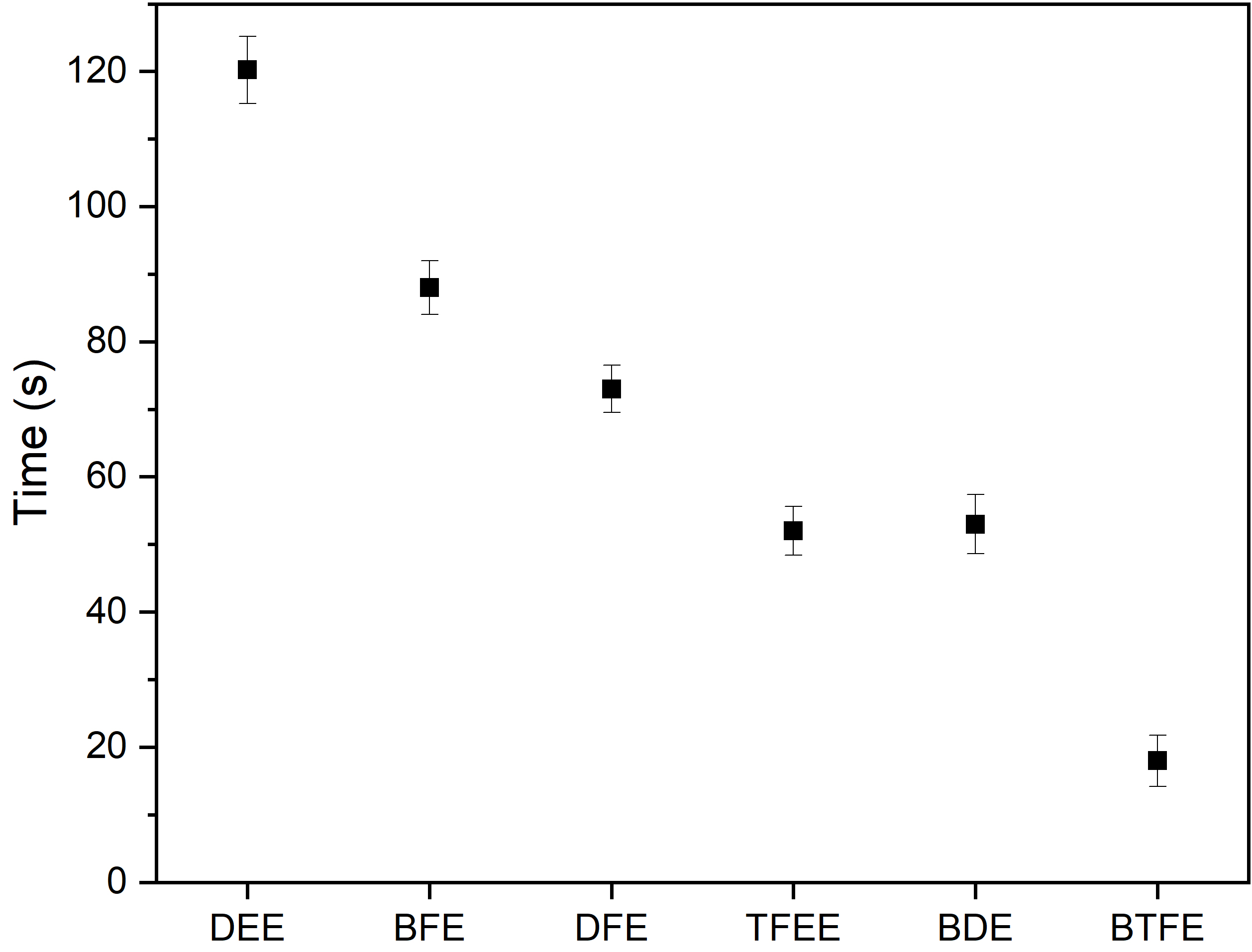


**Supplementary Figure 2.** The self-extinguishing time of different fluorinated solvents. Errors were caused by timekeeping.


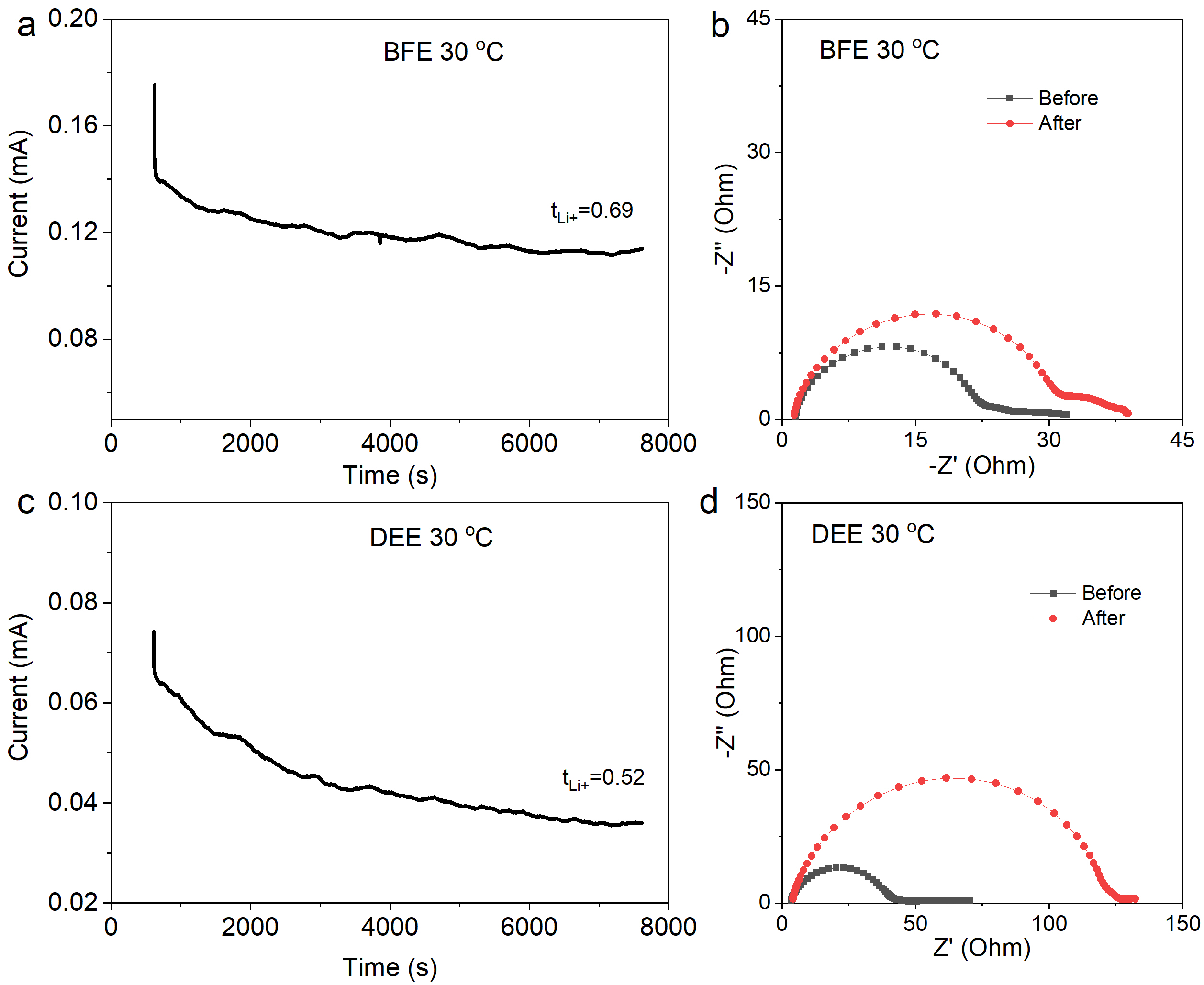


**Supplementary Figure 3.** Selected data for transference number calculations at 30 ℃ in coin cells. a, b) 5 mV polarization curves for the BFE and DEE electrolytes. Complex impedance plots before and after polarization used for t_Li_^+^ calculations of c) BFE, and d) DEE electrolyte.


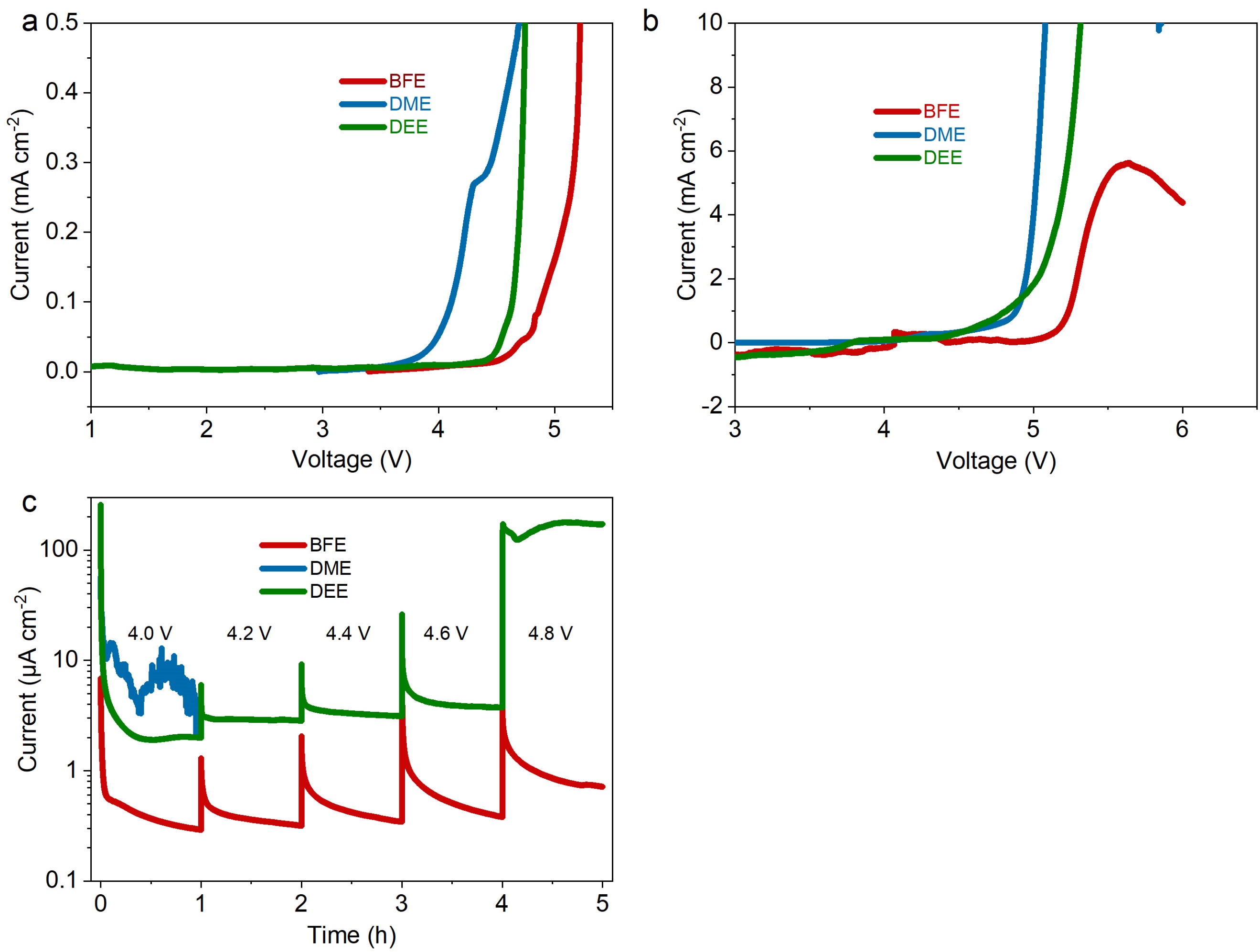


**Supplementary Figure 4.** Oxidation voltage measurements of different electrolytes. **a**, Linear sweep voltammetry (LSV) test using Li||Al coin cells in different electrolytes. **b**, LSV curves of Li||platinum (Pt) coin cells with different electrolytes. **c**, Potentiostatic polarization of Li||Al coin cells with increasing voltages in different electrolytes.


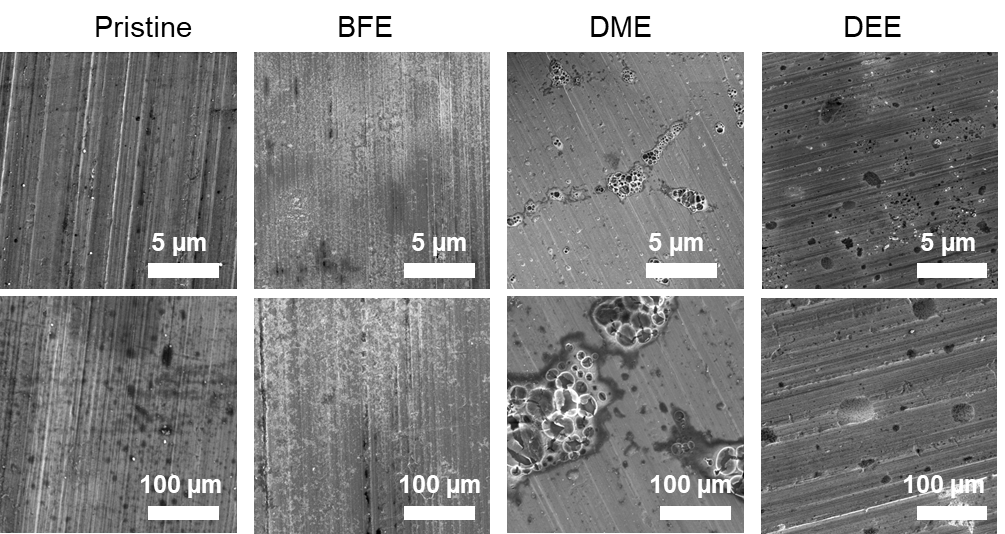


**Supplementary Figure 5.** Al corrosion test in different electrolytes via SEM after holding at 4.8 V for 5 hours at 30 ℃ using coin cells.


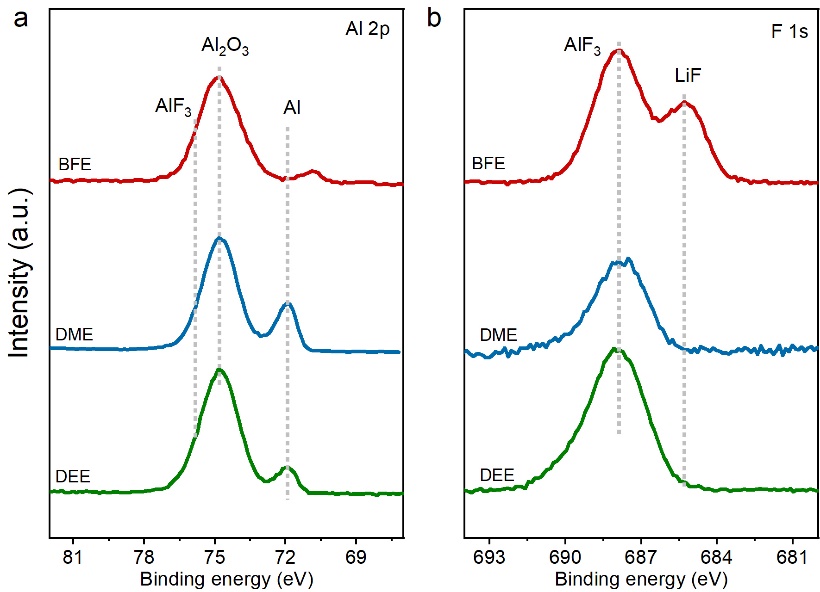


**Supplementary Figure 6.** Al 2p (a) and F 1s (b) spectra of Al foil after holding at 4.8 V for 5 hours using coin cells.


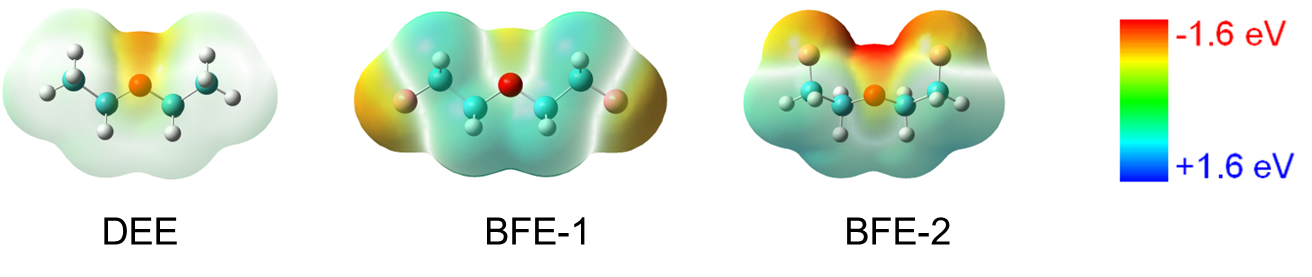


**Supplementary Figure 7.** Comparison of electrostatic potentials (ESP) between DEE and BFE. Spheres in white, pink, light blue and red color represent H, F, C, and O atoms, respectively.


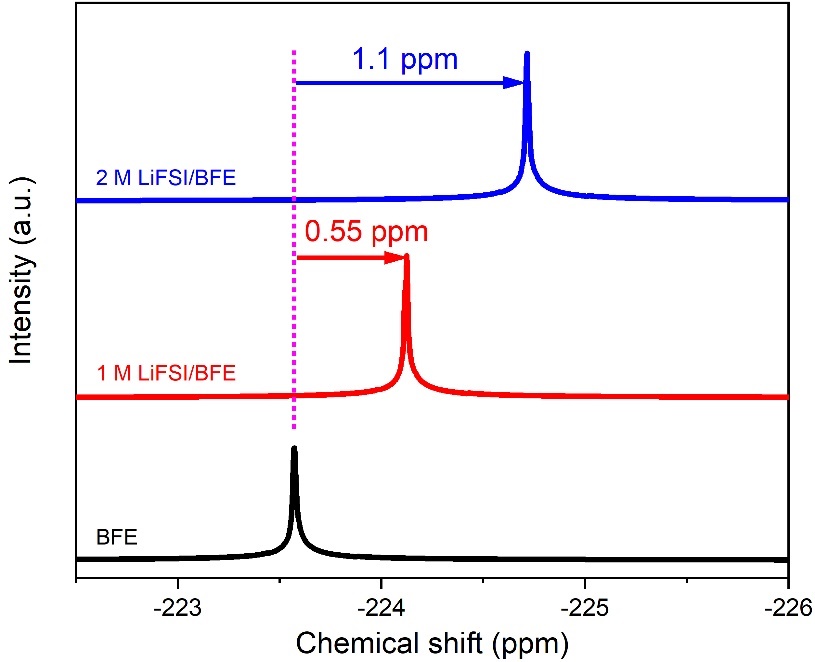


**Supplementary Figure 8.** ^19^F NMR of BFE molecules in pure solvent and electrolyte solutions with various LiFSI concentrations.


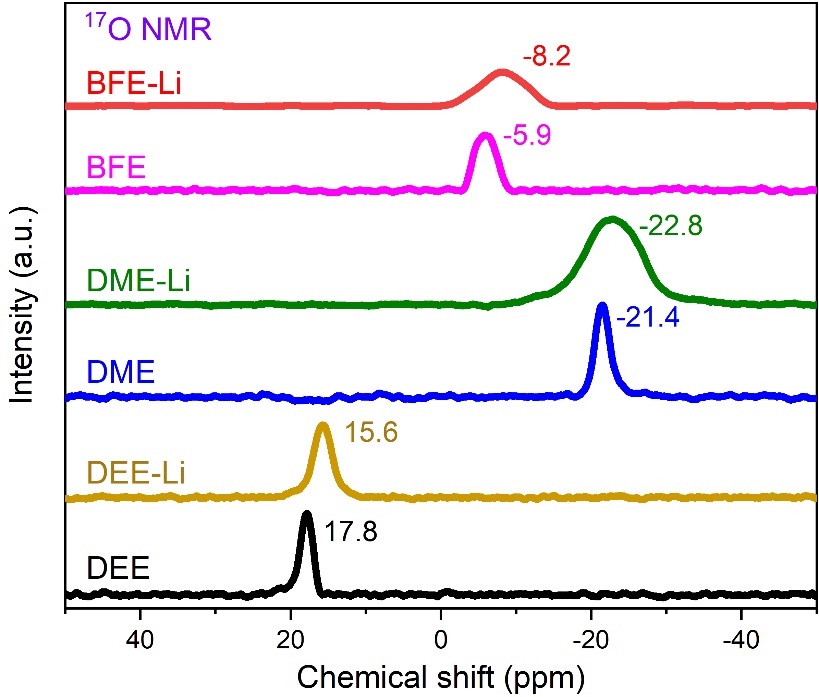


**Supplementary Figure 9.** ^17^O NMR of different ether solvents before and after dissolving 2 M LiFSI.


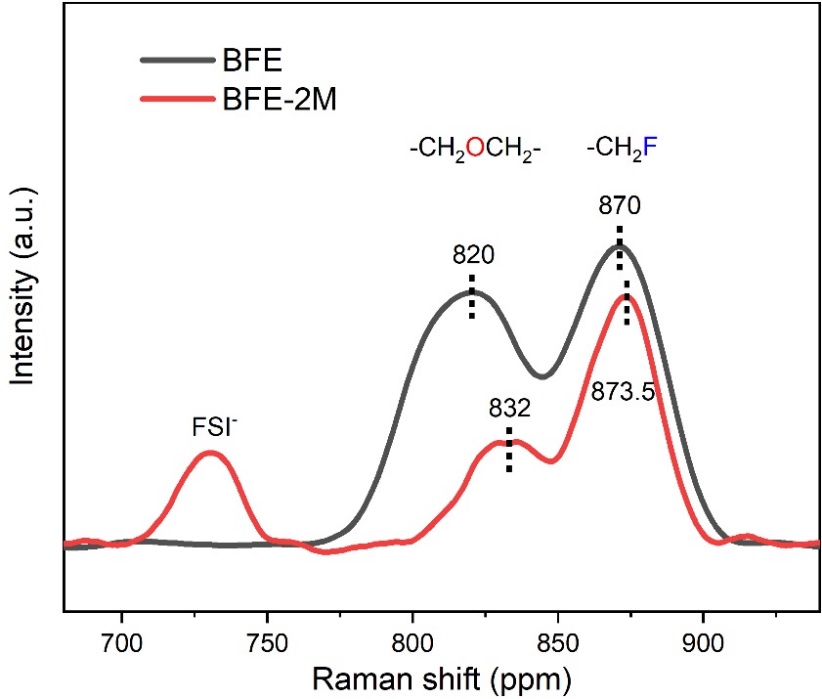


**Supplementary Figure 10.** The Raman spectra of BFE before and after dissolving 2 M LiFSI.


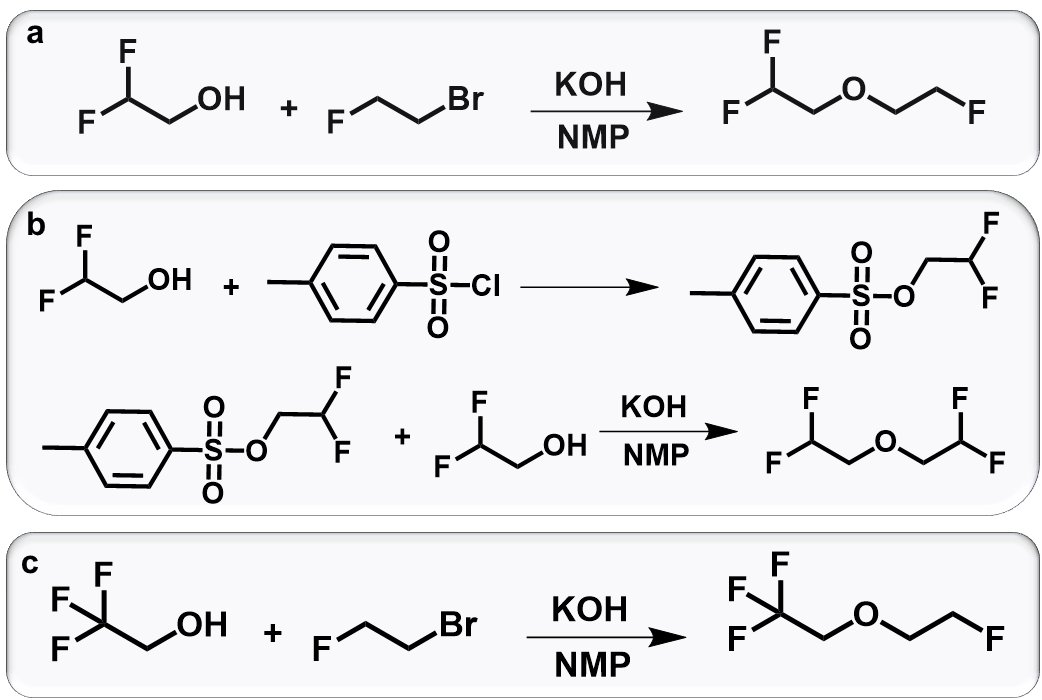


**Supplementary Figure 11.** The synthesis of 2,2-difluoroethyl-2-fluoroethyl ether (DFE, a), bis(difluoroethyl) ether (BDE, b) and 2,2,2-trifluoroethyl-2-fluoroethyl ether (TFFE, c).


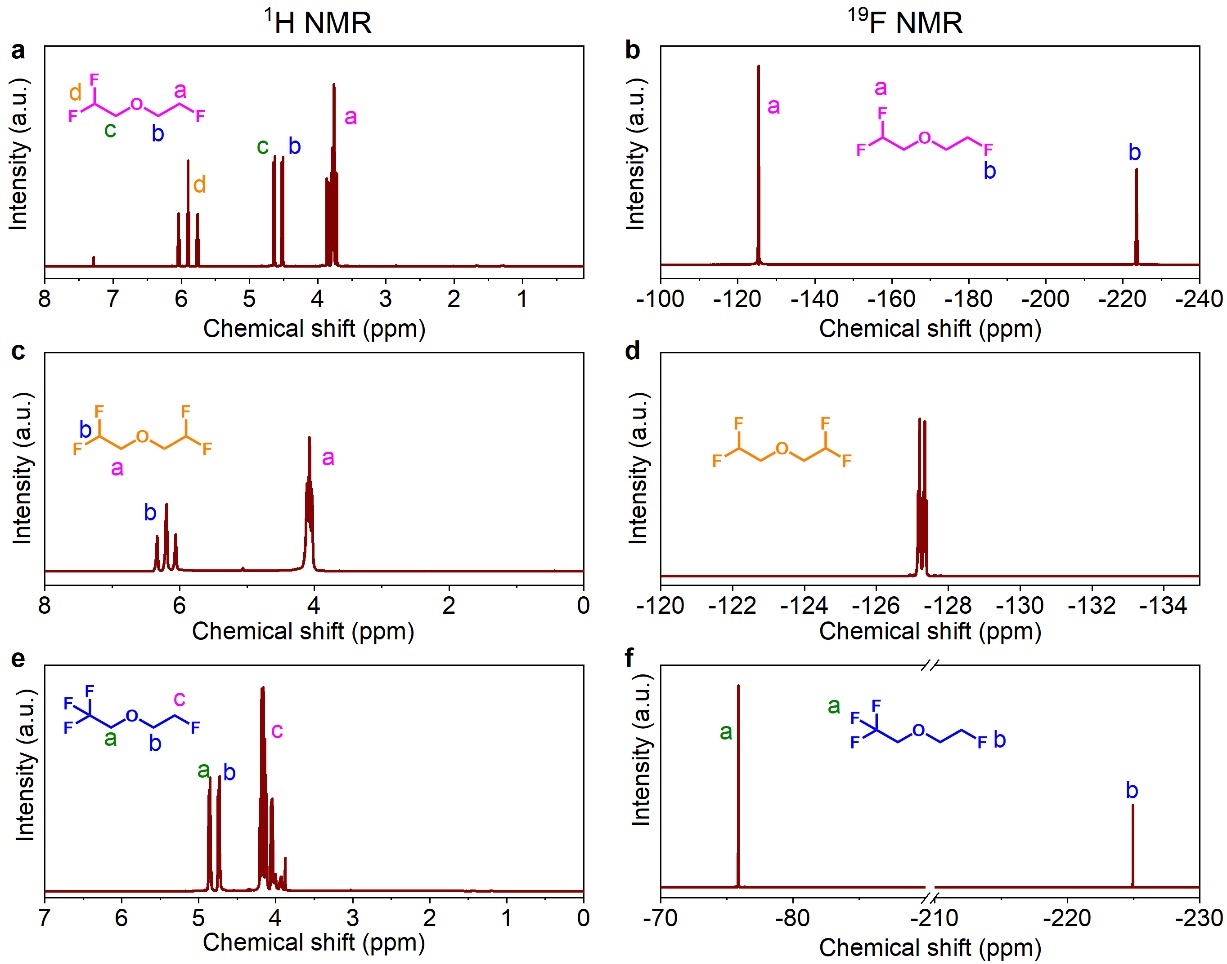


**Supplementary Figure 12.** ^1^H and ^19^F NMR of DFE (a, b), BDE (c, d) and TFFE (e, f).


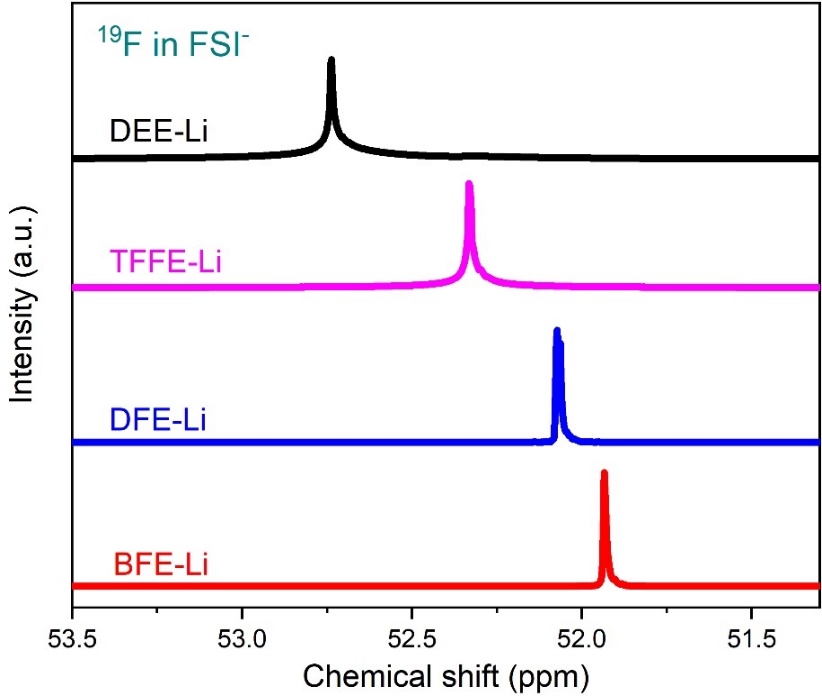


**Supplementary Figure 13.** ^19^F NMR in FSI^-^ anions of different electrolytes with 2 M LiFSI.


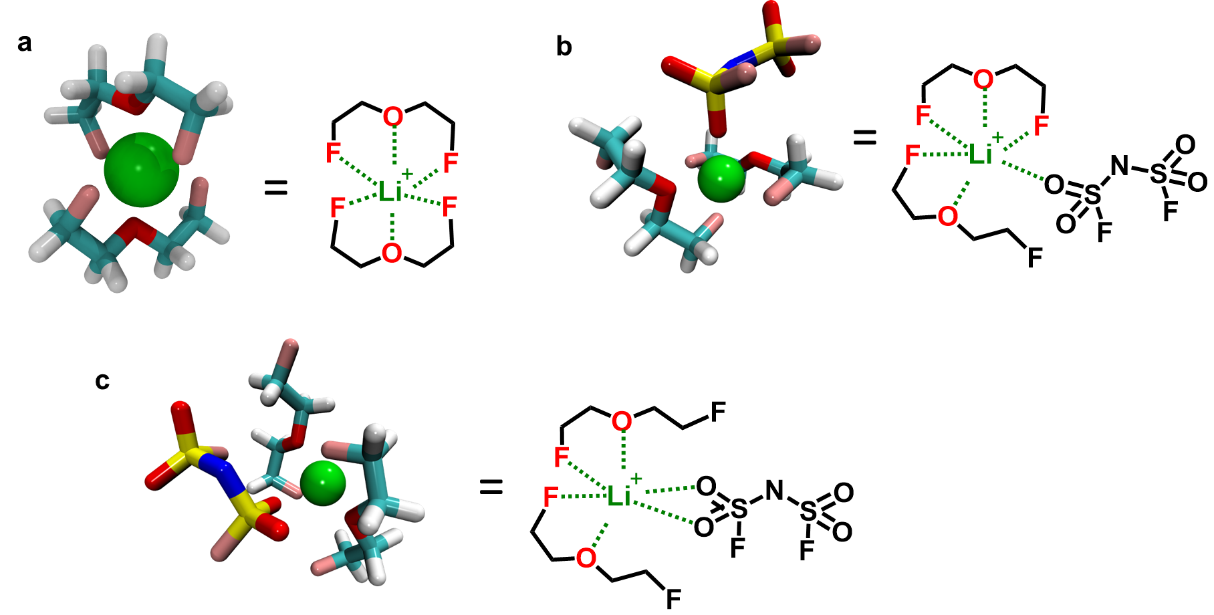


**Supplementary Figure 14.** Solvation clusters extracted from the MD simulation snapshot of the 2 M LiFSI/BFE electrolyte. **a**, Solvent separated ion pairs, represent 60% of the total lithium in the electrolyte solution. **b**, **c**) Contact ion pairs with different coordination formats.


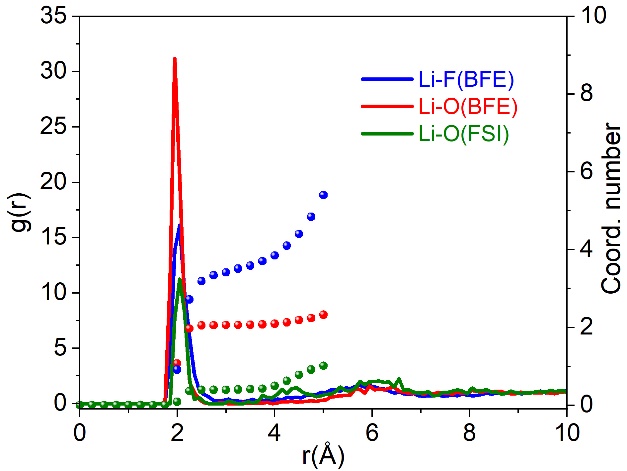


**Supplementary Figure 15.** Radical distribution functions comparison of Li-O (BFE), Li-F (BFE), and Li-O (FSI) pairs for BEE electrolytes and the corresponding coordination numbers.


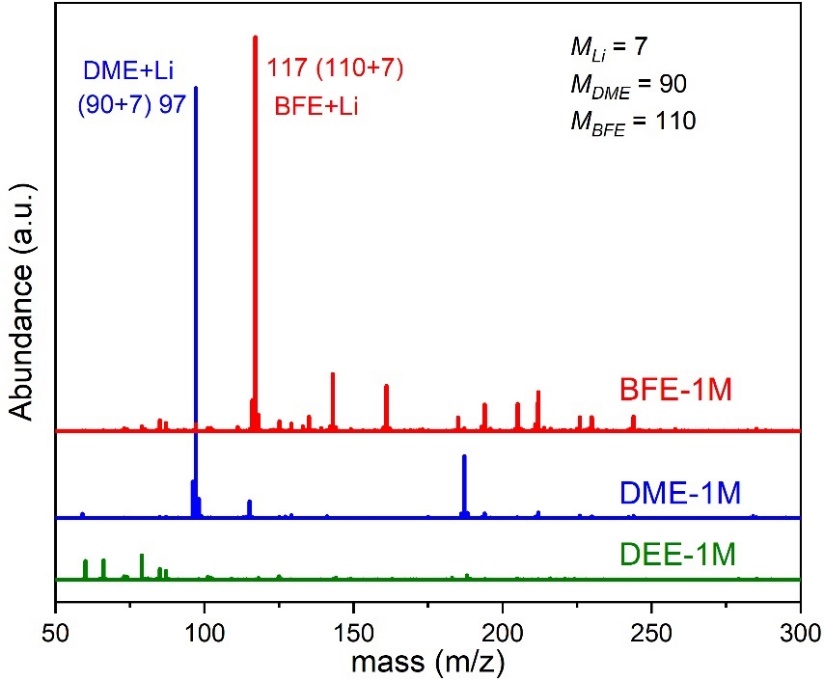


**Supplementary Figure 16.** Electrospray ionization mass spectrometry (ESI-MS) of different electrolytes.


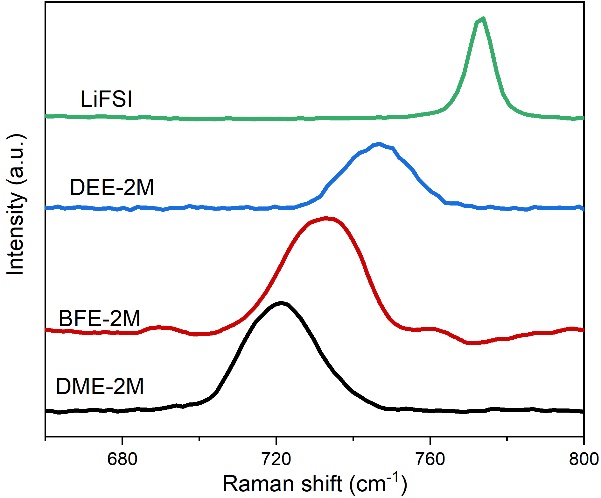


**Supplementary Figure 17.** Raman spectrum of different electrolytes at 30 ℃.


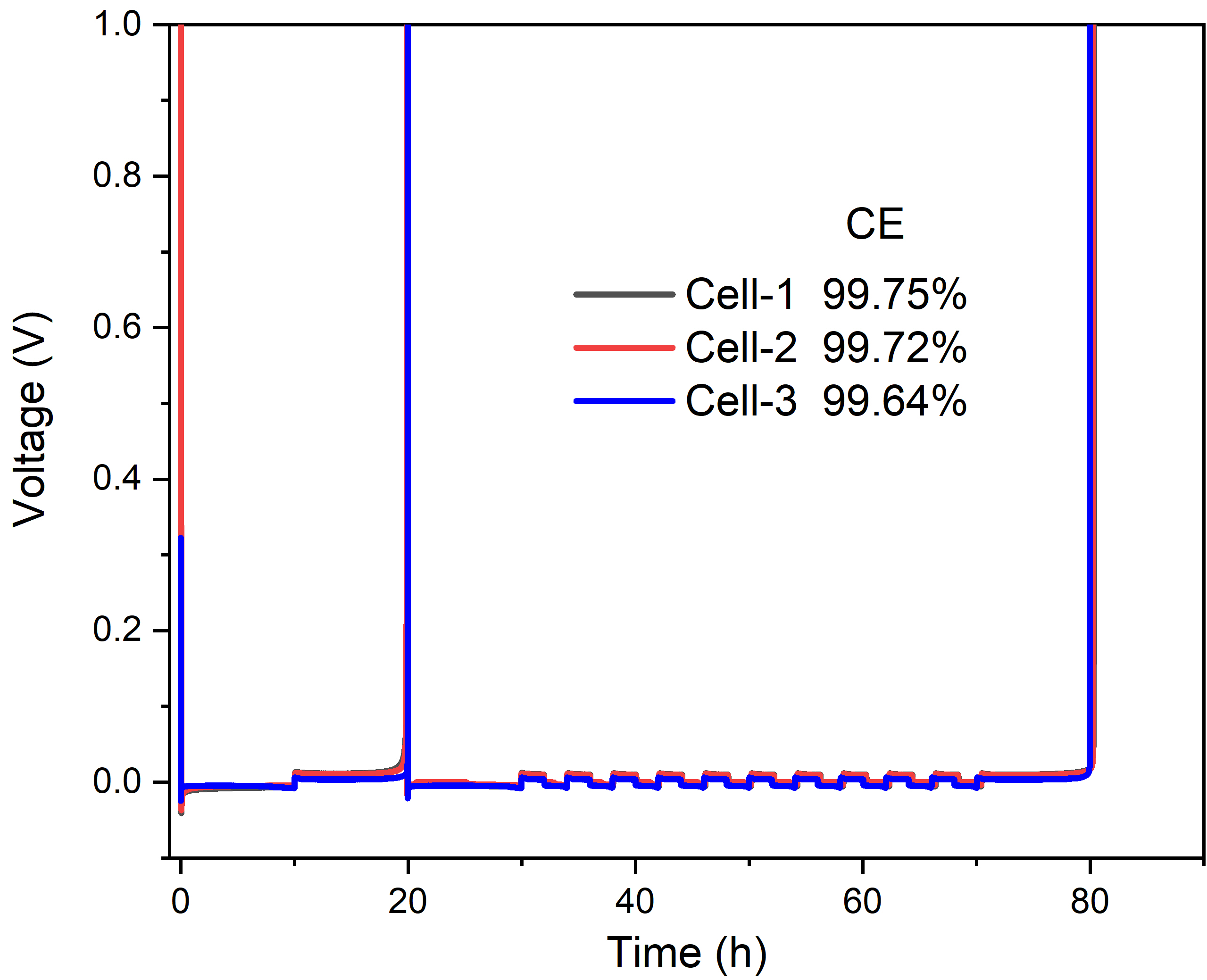


**Supplementary Figure 18.** Li plating/stripping CEs of BFE electrolyte evaluated via Aurbach’s measurement using Li||Cu cells at 0.5 mA cm^-2^ with the capacity of 5 mAh cm^-2^.


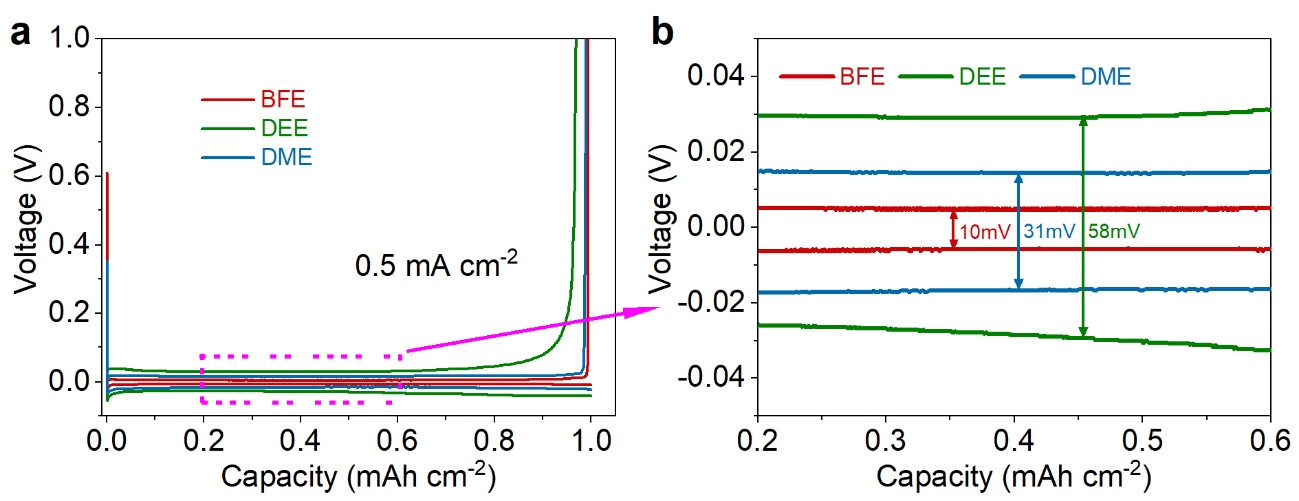


**Supplementary Figure 19.** Charge/discharge curves of Li||Cu coin cells in different electrolytes at 0.5 mA cm^-2^ (30 ℃).


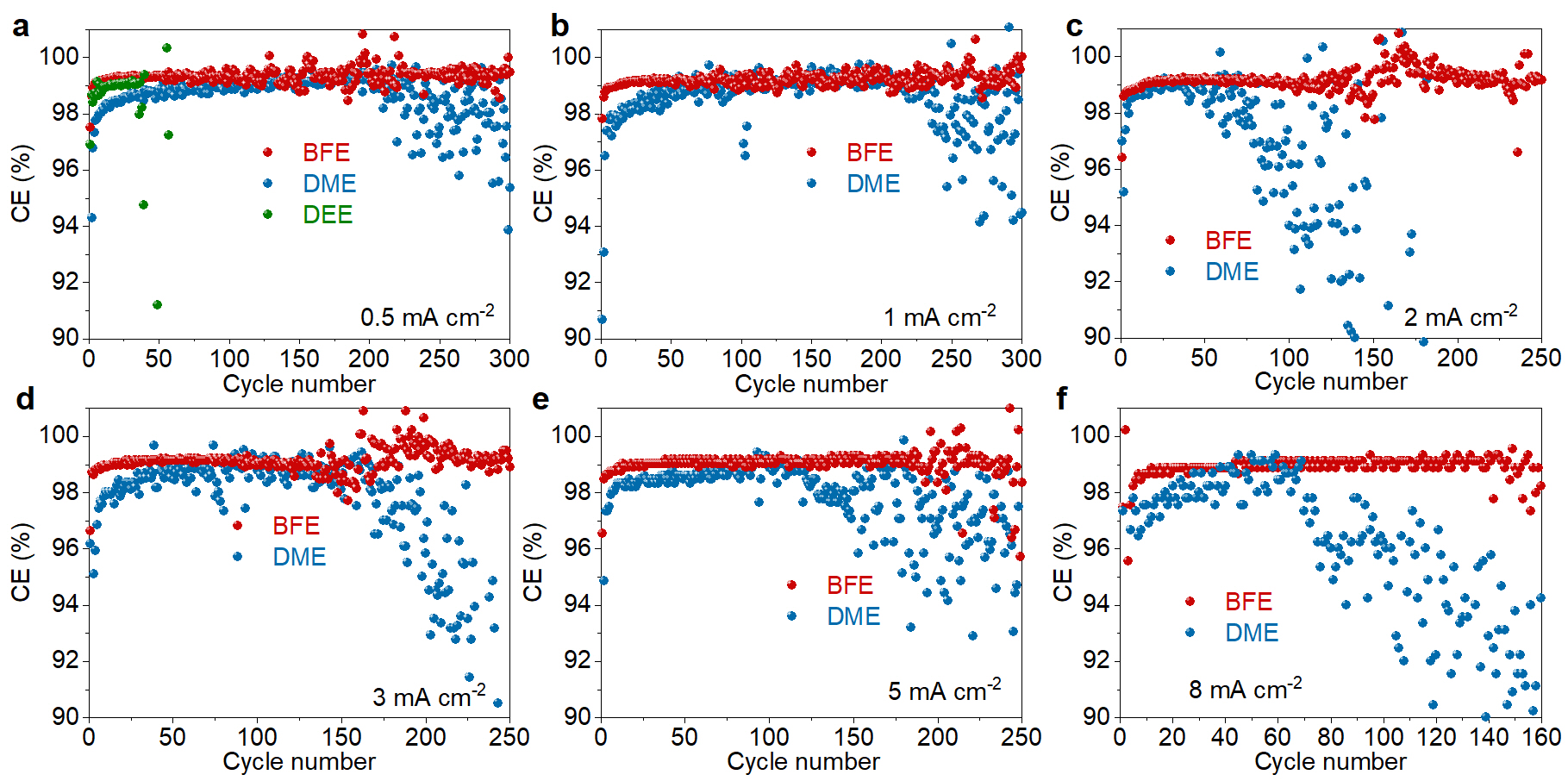


**Supplementary Figure 20.** Cycling performance of Li||Cu cells in different electrolytes at 0.5 (a), 1.0 (b), 2.0 (c), 3.0 (d), 5.0 (e) and 8.0 mA cm^-2^ (f), respectively (coin cells at 30 ℃).


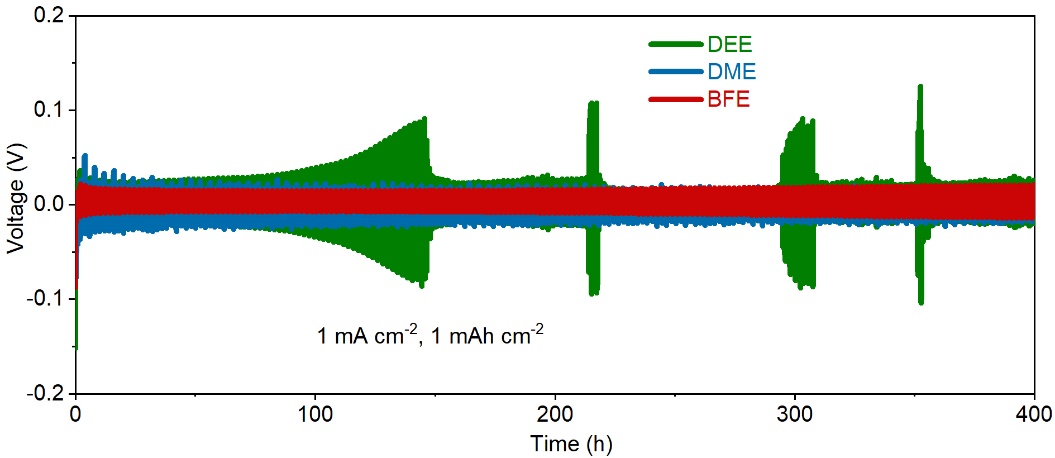


**Supplementary Figure 21.** Long cycling test of Li||Li symmetric coin cells at 1 mA cm^-2^, 1 mAh cm^-2^ in different electrolytes at 30 ℃.


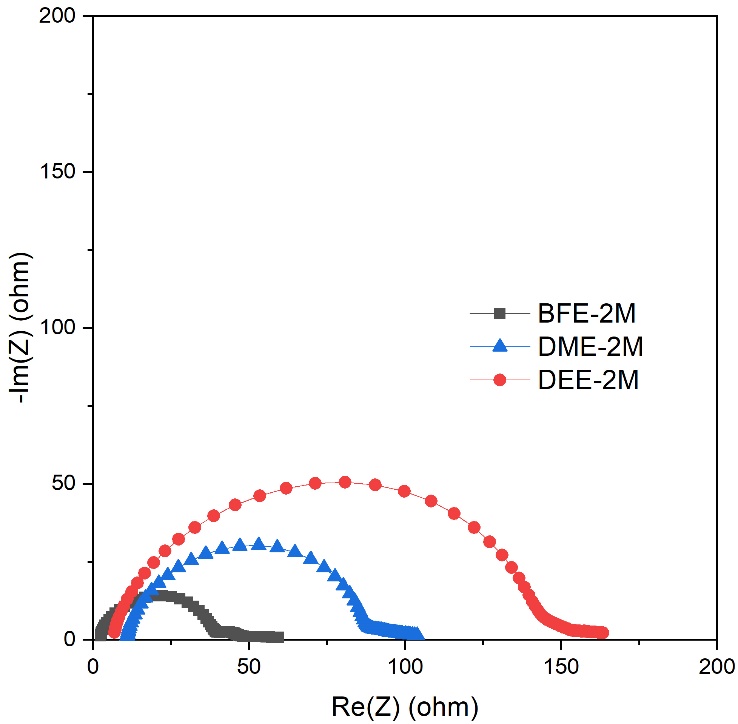


**Supplementary Figure 22.** Electrochemical impedance spectra of Li||Li symmetric cells after cycling in different electrolytes at 30 ℃. Cells were tested after 200 cycles.


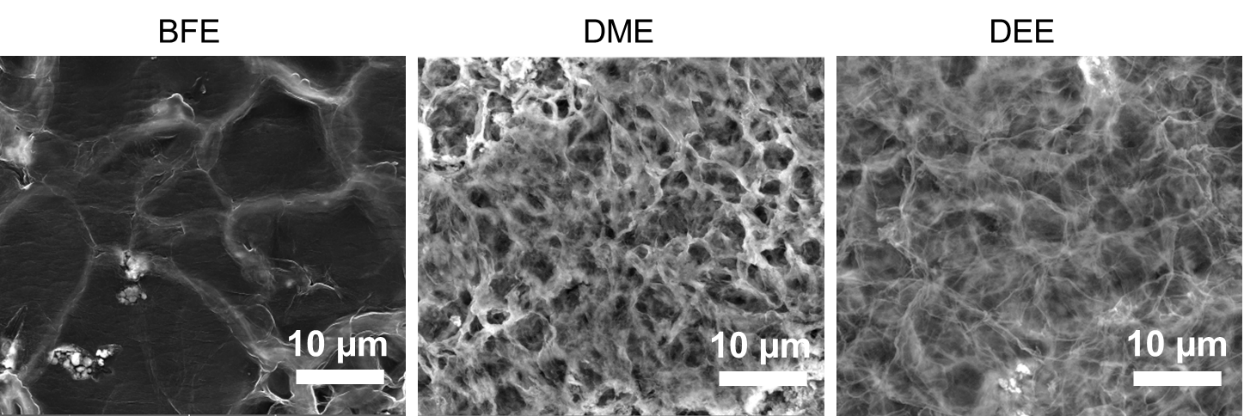


**Supplementary Figure 23.** SEM images of Li metal after 250 cycles using Li||Li symmetric cells in BFE and DEE electrolytes at the current density of 0.5 mA cm^-2^ (30 ℃).


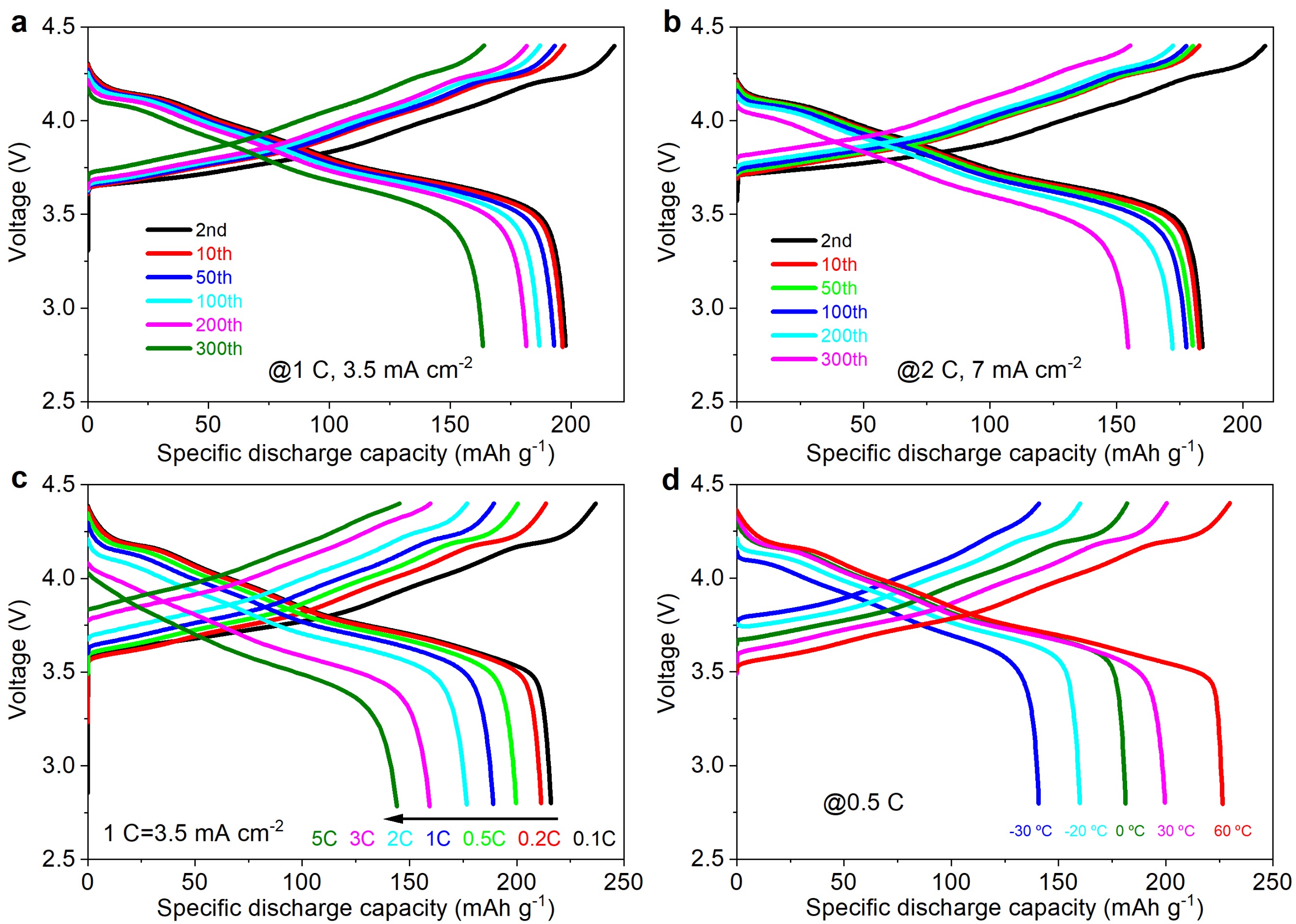


**Supplementary Figure 24.** Charge/discharge curves of Li||NCM811 coin cells using BFE electrolyte at 3.5 (a) and 7.0 mA cm^-2^ (b). (c) Rate performance of Li||NCM811 cell in BFE electrolyte. Cells were tested at 30 ℃. (d) Charge/discharge curves Li||NCM811 cell in BFE electrolyte at different temperatures.


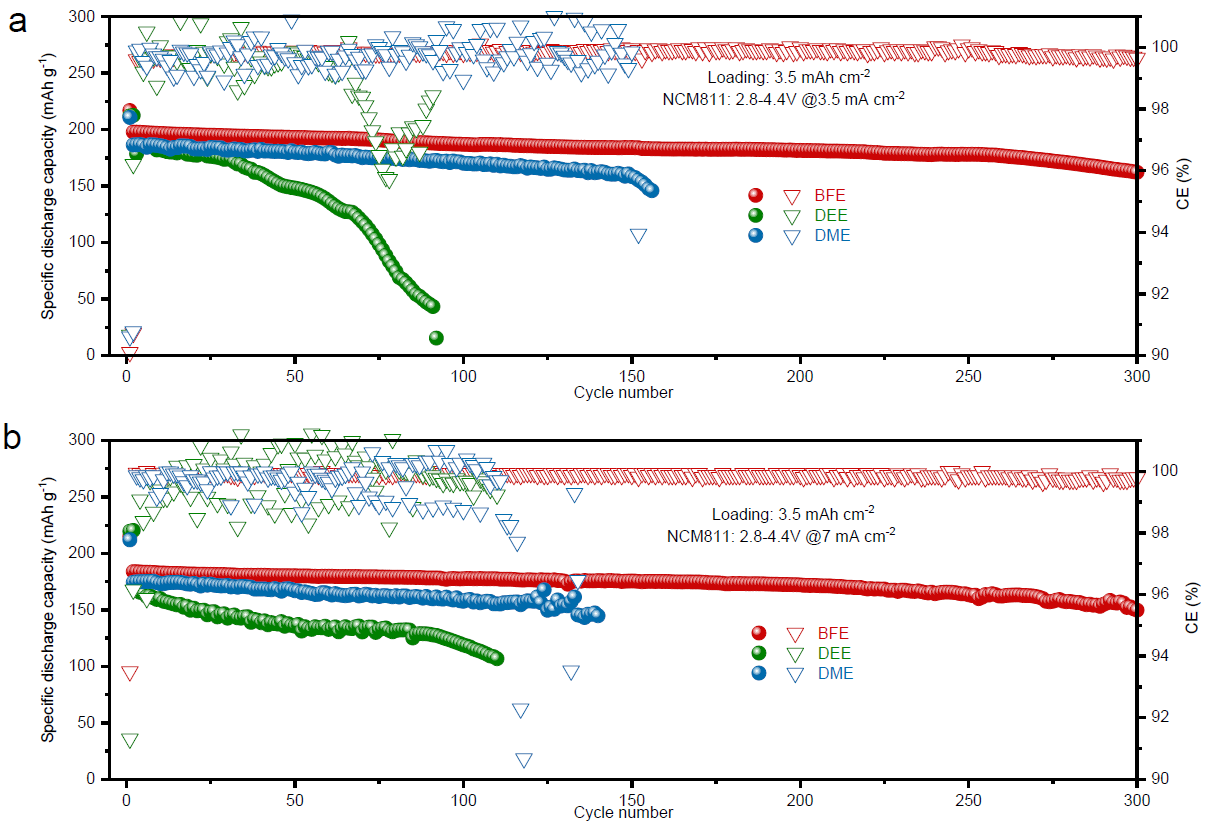


**Supplementary Figure 25.** Cycling performance of Li||NCM811 coin cells at 30 ℃ in different electrolytes at 3.5 (a) and 7 mAh cm^-2^ (b).


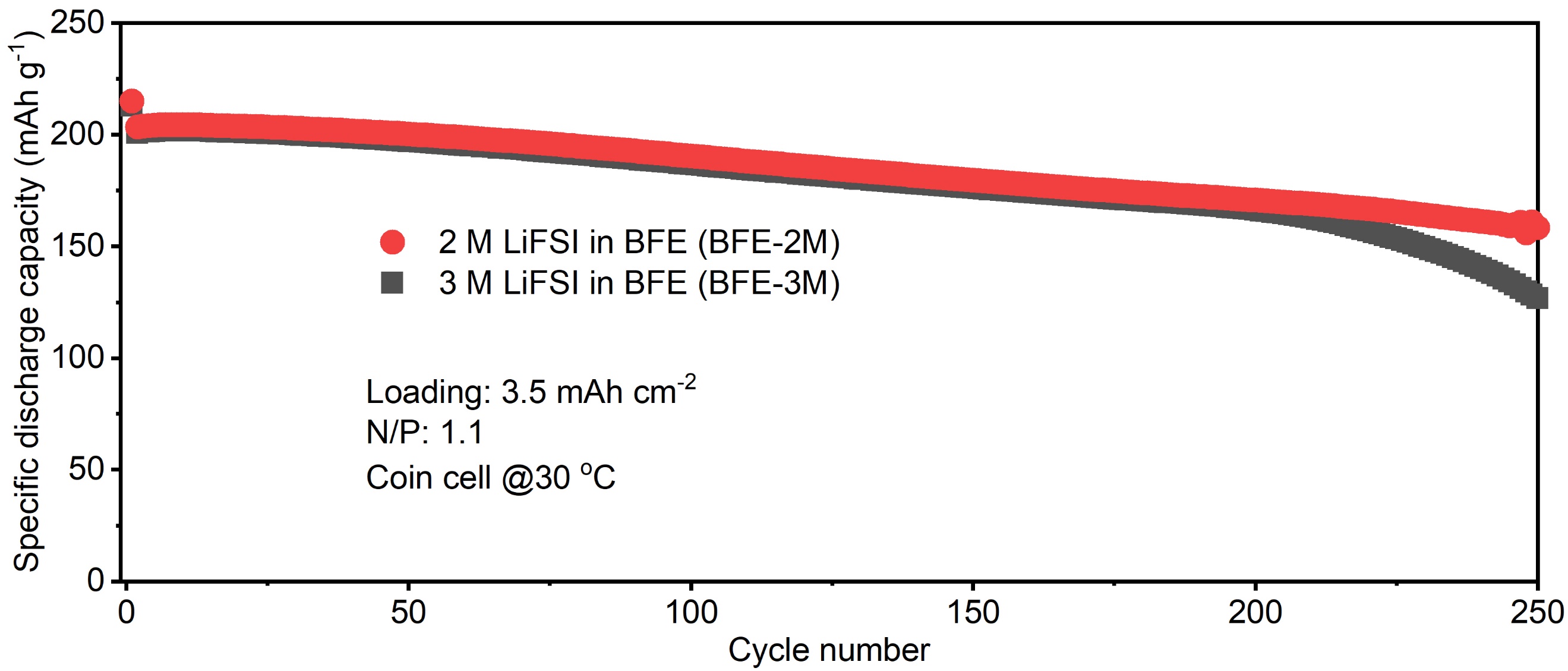


**Supplementary Figure 26.** The long cycling performance of Li||NCM811 coin cells with BFE-2M and BFE-3M electrolytes at 1 mA cm^-2^.


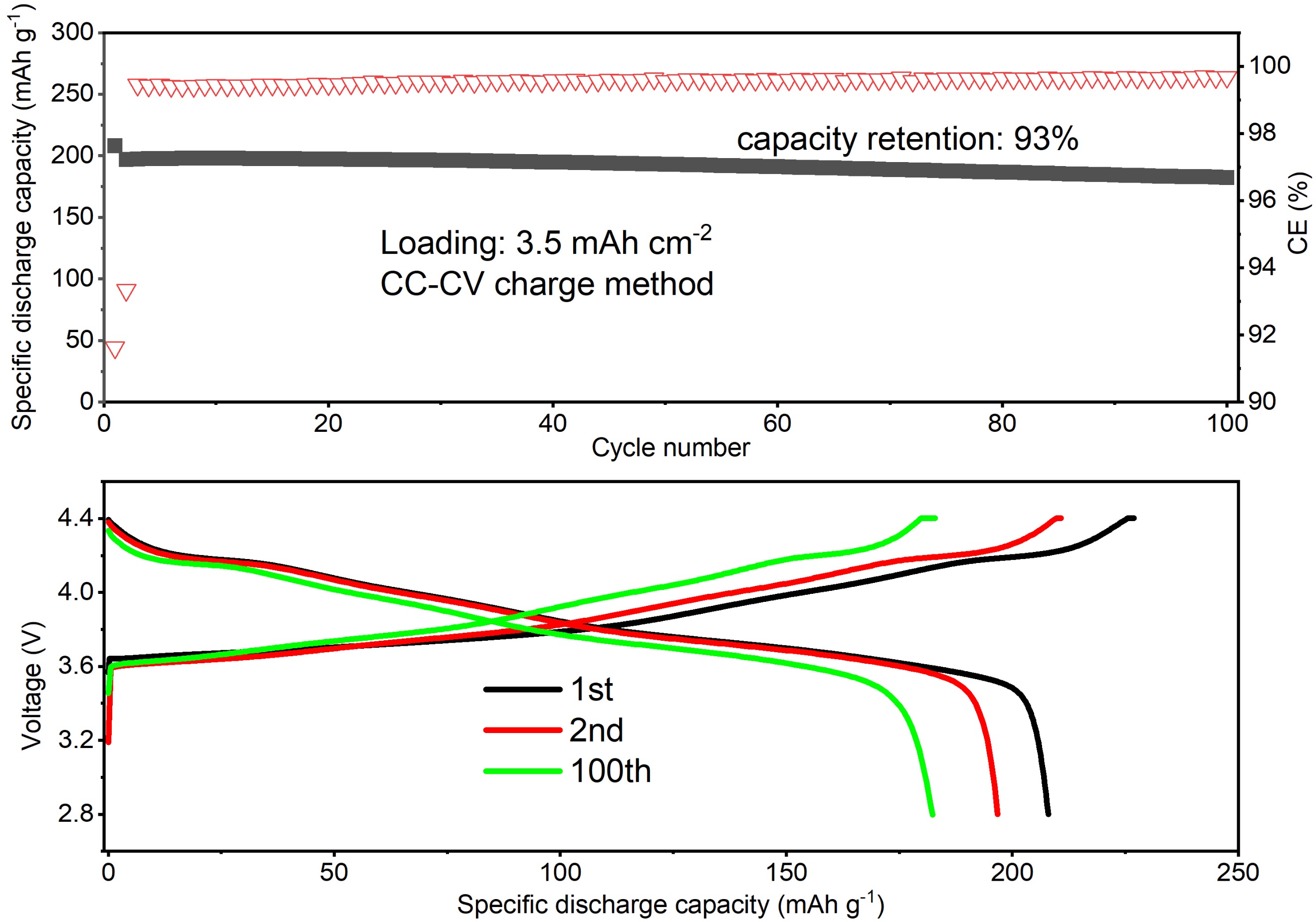


**Supplementary Figure 27.** The long cycling performance and corresponding charge/discharge profiles of Li||NCM811 coin cell at 30 ℃ in BFE electrolyte using CC-CV method at 1 mA cm^-2^.


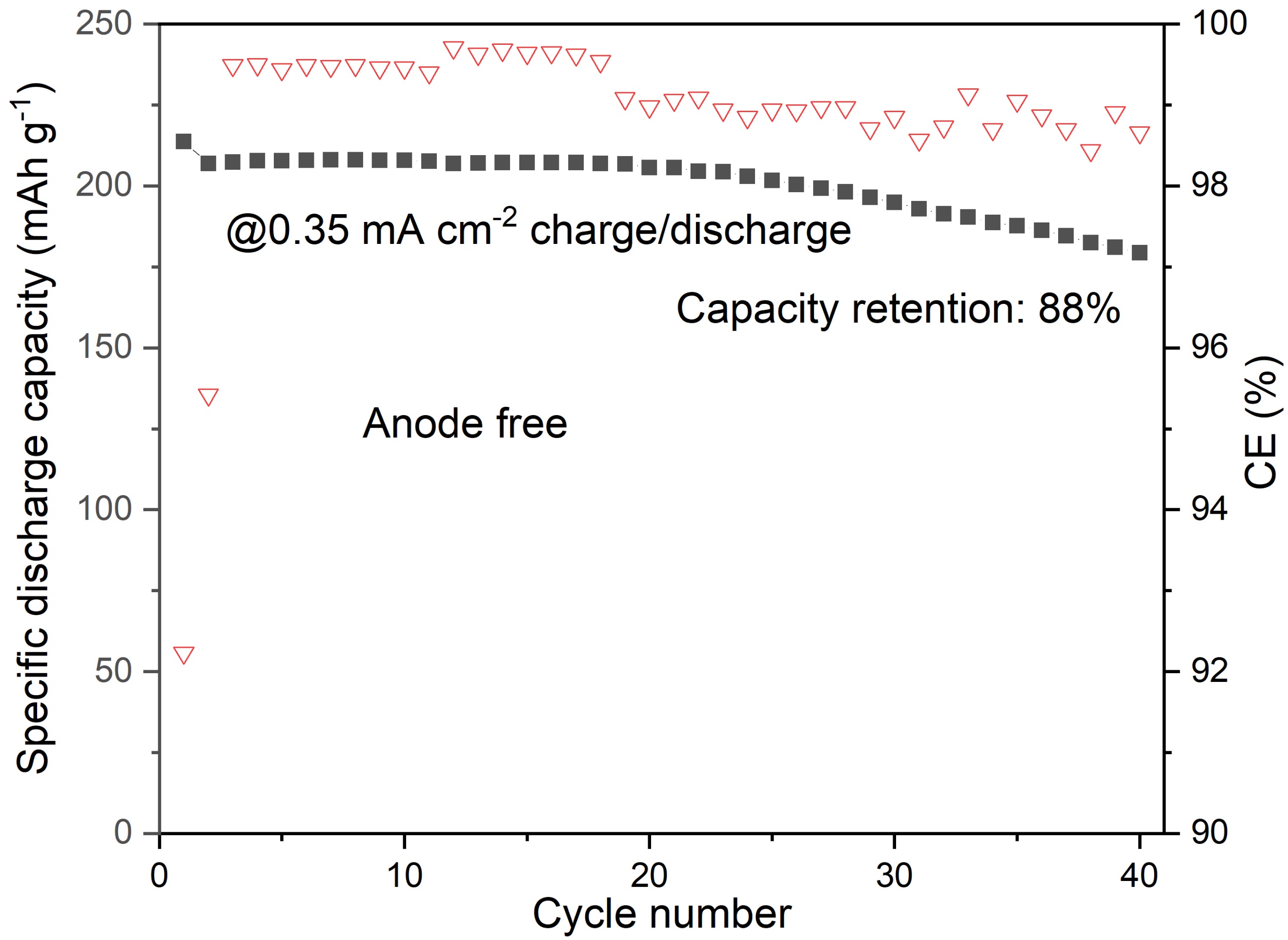


**Supplementary Figure 28**. The cycling performance of anode-free coin cell in BFE electrolyte at 30 ℃. Current density: 0.35 mA cm^-2^.


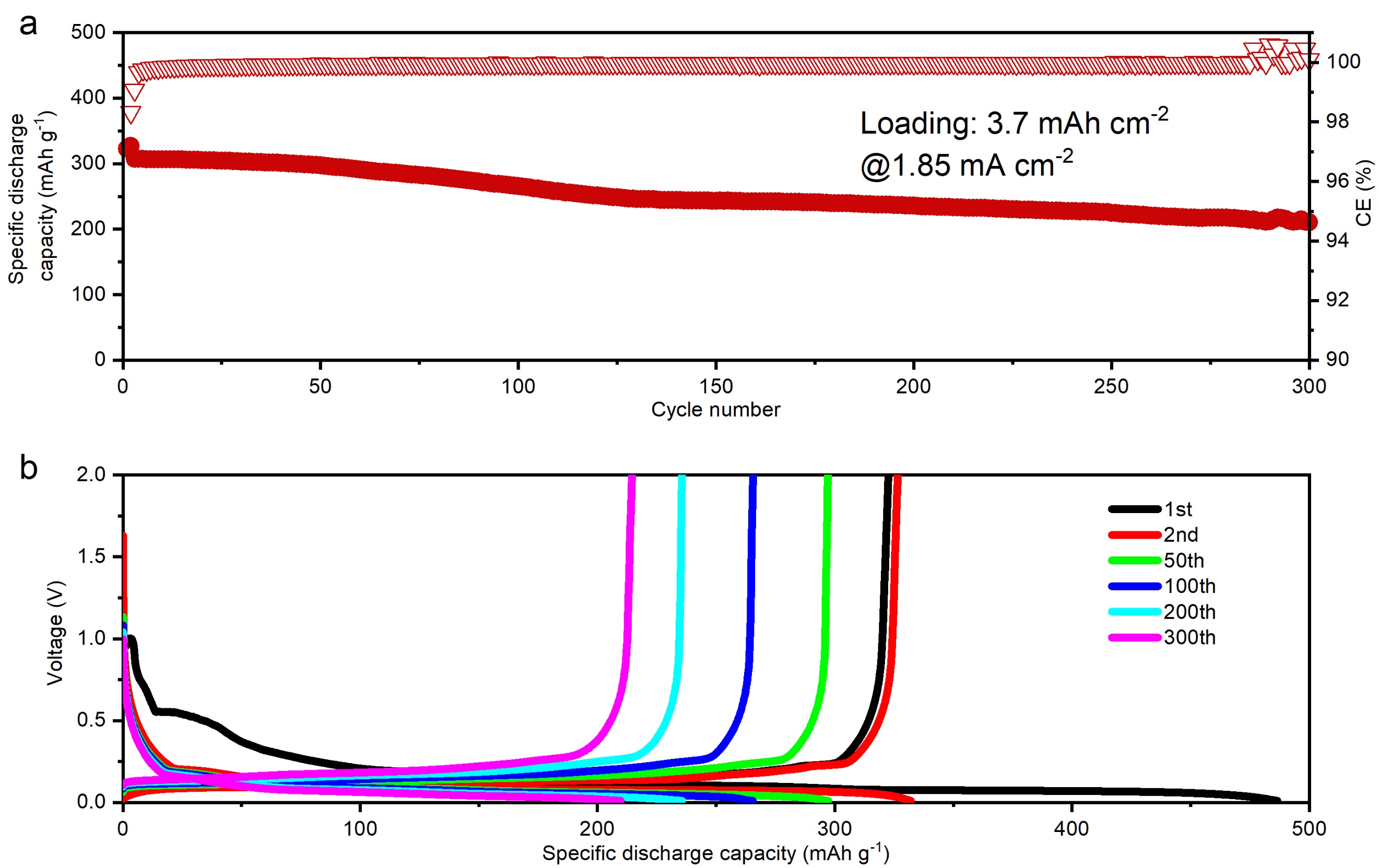


**Supplementary Figure 29.** Cycling performance (a) and charge/discharge curves (b) of Li||Graphite coin cell in BFE electrolyte at 30 ℃.


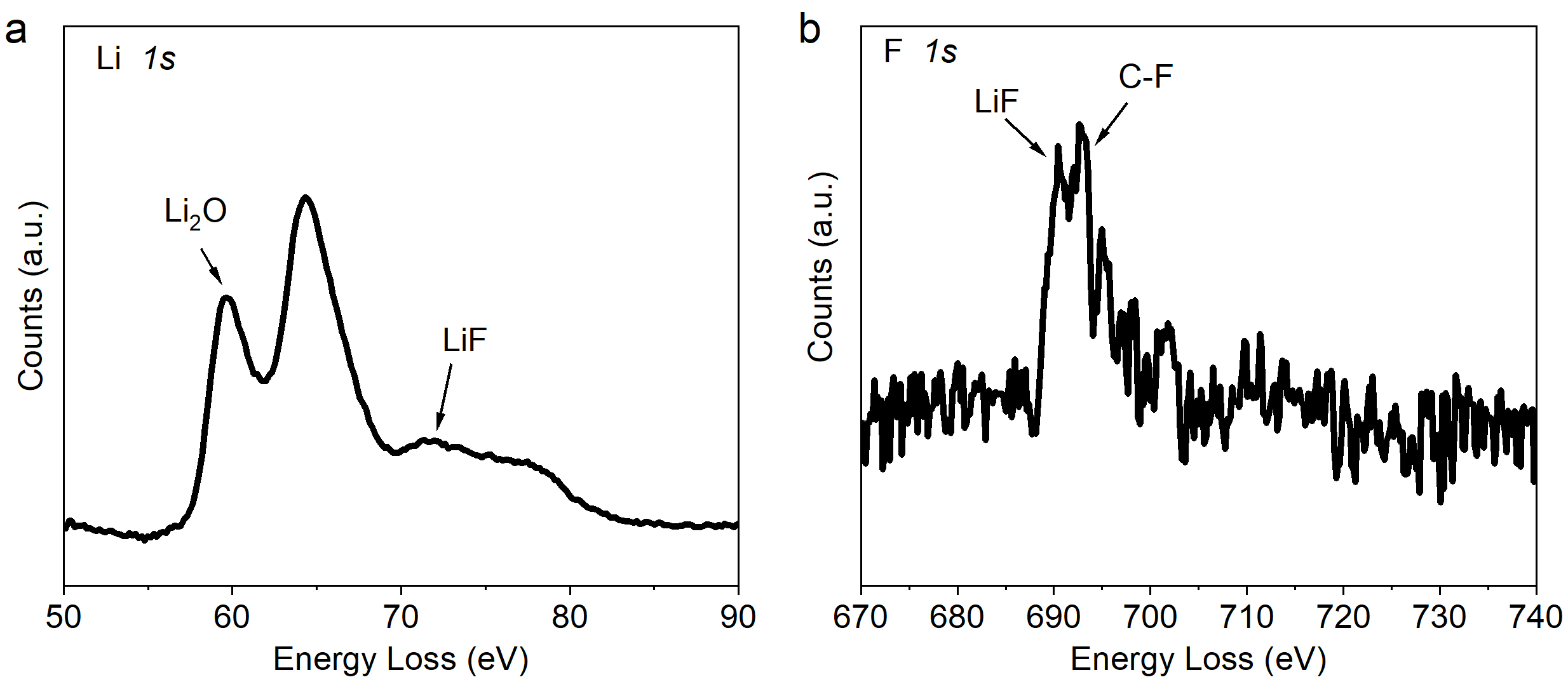


**Supplementary Figure 30**. EELS spectra of Li metal in BFE electrolyte. (The Li||Cu coin cell

was discharged with the capacity of 0.25 mAh cm^-2^ at 0.5 mA cm^-2^).


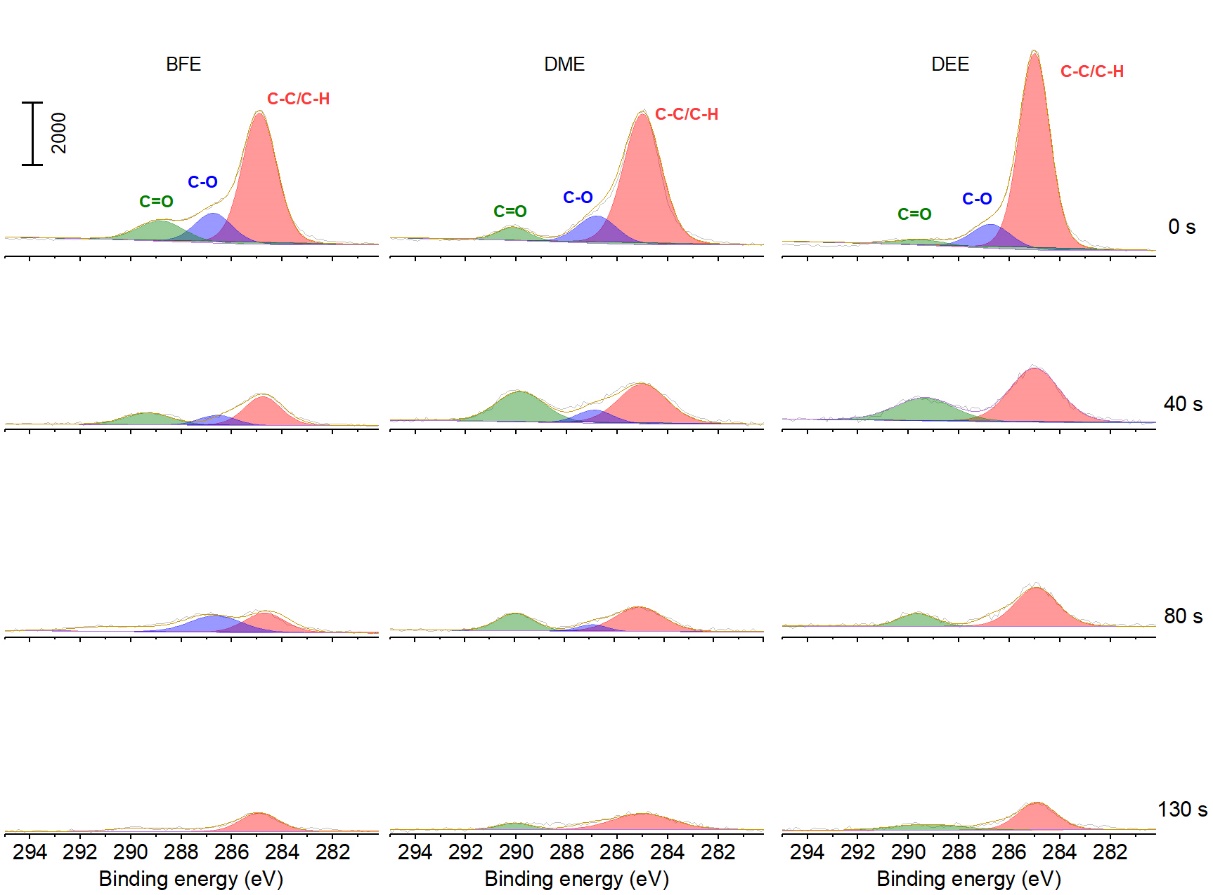


**Supplementary Figure 31.** C1s XPS spectra with etching depth profiles of the cycled Li metals in different electrolytes (BFE, DME and DEE). Li||Li coin cells were cycled with the capacity of 5 mAh cm^-2^ at the current density of 0.5 mA cm^-2^ for 5 cycles.


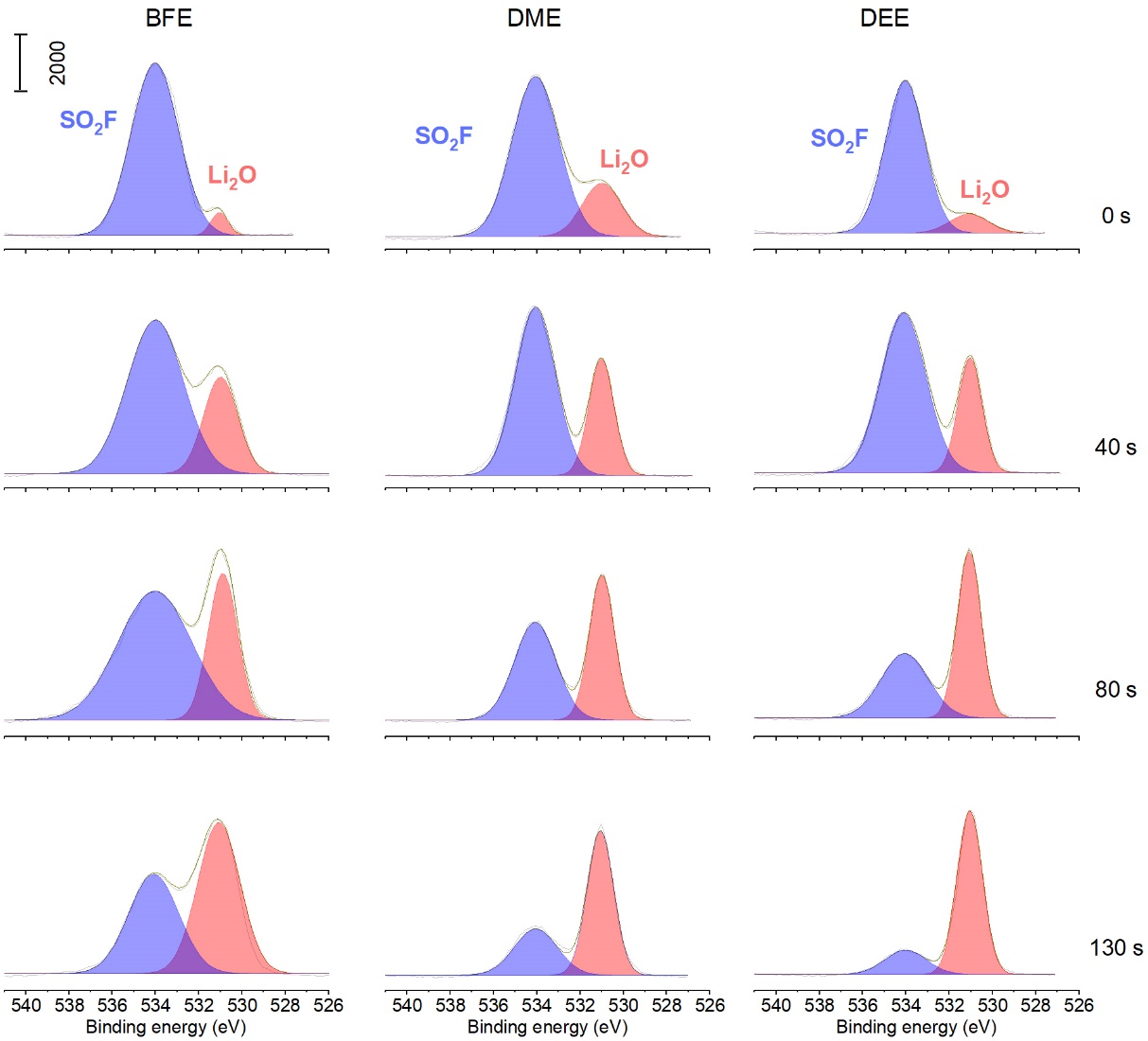


**Supplementary Figure 32.** O1s XPS spectra with etching depth profiles of the cycled Li metals in different electrolytes (BFE, DME and DEE). Li||Li coin cells were cycled with the capacity of 5 mAh cm^-2^ at the current density of 0.5 mA cm^-2^ for 5 cycles.


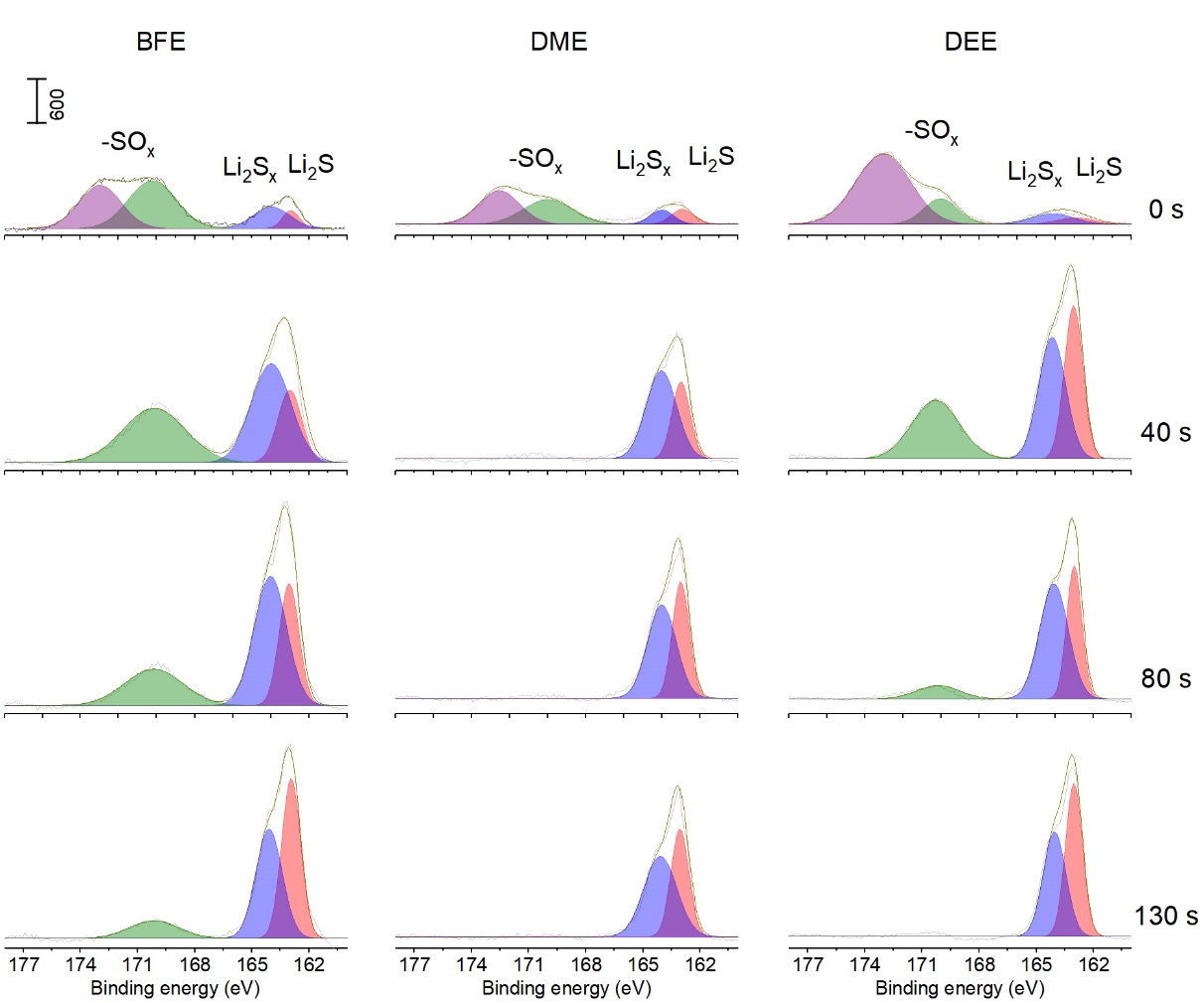


**Supplementary Figure 33.** S2p XPS spectra with etching depth profiles of the cycled Li metals in different electrolytes (BFE, DME and DEE). Li||Li coin cells were cycled with the capacity of 5 mAh cm^-2^ at the current density of 0.5 mA cm^-2^ for 5 cycles.


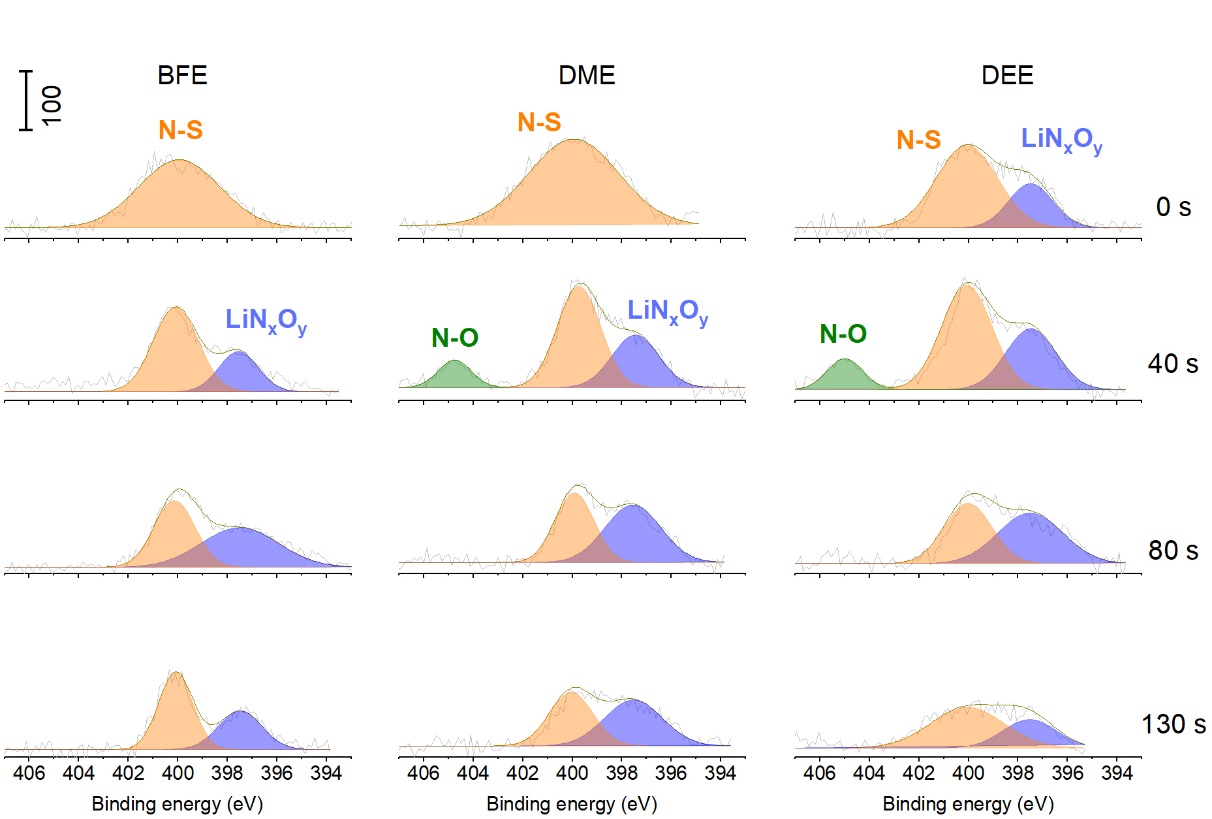


**Supplementary Figure 34.** N1s XPS spectra with etching depth profiles of the cycled Li metals in different electrolytes (BFE, DME and DEE). Li||Li coin cells were cycled with the capacity of 5 mAh cm^-2^ at the current density of 0.5 mA cm^-2^ for 5 cycles.


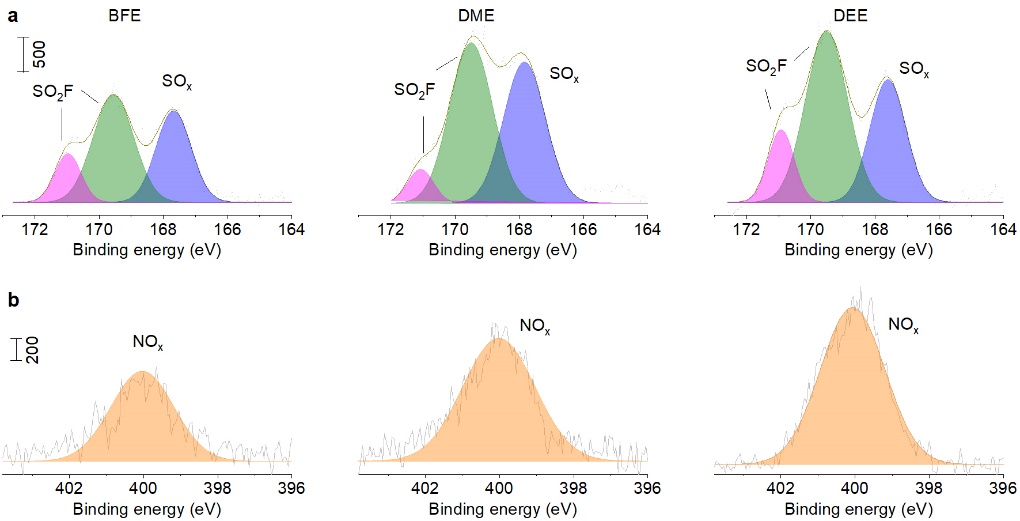


**Supplementary Figure 35.** S2p (a) and N1s (b) XPS spectra of the cycled NCM811 cathodes in different electrolytes (BFE, DME and DEE). The Li||NCM811 coin cells were cycled between 2.8 and 4.4 V for 50 cycles at 30 ℃, and the cells were disassembled after discharging to 2.8 V.


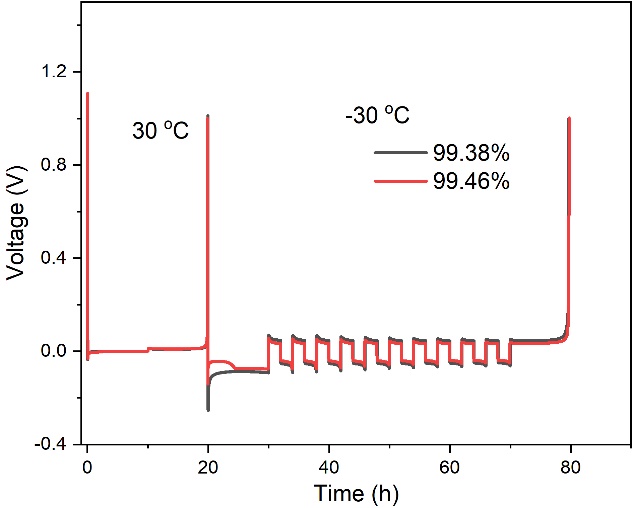


**Supplementary Figure 36**. Li plating/stripping CEs evaluated via Aurbach’s measurement at -30 ^o^C using coin cells.


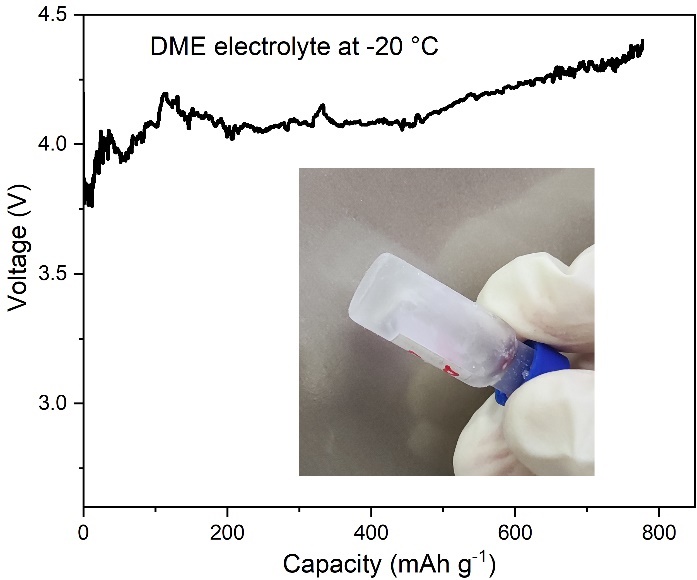


**Supplementary Figure 37.** Charge curve of Li||NCM811 coin cell in DME electrolyte at -20 ℃, and optical picture of DME electrolyte at -20 ℃.

**Supplementary Table 1**. Physical properties of different ether solvents

| Compound | ^a^HOMO (eV) | ^a^LUMO (eV) | Boiling point (^o^C) | Density  (g mL^-1^) | Viscosity  (cp) |
| --- | --- | --- | --- | --- | --- |
| DEE | -6.75 | 2.46 | 34.5 | 0.71 | 0.25 |
| DME | -6.82 | 2.31 | 84 | 0.87 | 0.3 |
| BFE | -7.23 | 2.24 | 127 | 0.98 | 0.32 |
| DFE | -7.48 | 1.94 | 118 | 1.1 | 0.3 |
| BDE | -7.83 | 1.61 | 104 | 1.18 | 0.28 |
| TFFE | -7.73 | 1.8 | 90 | 1.3 | - |
| BTFE | -8.8 | 1.32 | 63 | 1.4 | - |
| ^a^From DFT calculation. | | | | | |

**Supplementary Table 2.** The specifications of the Li||NCM811 pouch cell

| The specifications of the Li\|\|NCM811 pouch cell | | |
| --- | --- | --- |
| Cell component | Specification | Parameters |
| cathode  (NCM 811) | Active material mass loading (each side) | 18.7 mg cm^-2^ |
|  | Active material content | 96% |
|  | Number of electrodes | 4 |
|  | Dimension (6*3.3 cm*4) | 79.2 cm^-2^ |
|  | Specific discharge capacity | 218 mAh g^-1^, @ 0.4 mA cm^-2^ (2.8-4.4V) |
|  | Total mass | 1542.7 mg |
| Anode (Li) | 40 um (each side) | 2.1 mg cm^-2^ |
|  | Mass | 166.32 mg |
| Al foil | 12 um | 3.24 mg cm^-2^ |
|  | Mass | 128.3 g |
| Cu foil (copper mesh) | 8 um copper mesh | 3 mg cm^-2^ |
|  | Mass | 178.2 mg |
| Separator | 20 um | 1.14 mg cm^-2^ |
|  | Mass | 90.3 mg |
| Electrolyte | 2.4 g Ah^-1^ | 768 mg |
| Total mass |  | 2873.8 mg |
| Total energy |  | 1.226 Wh |
| Specific energy |  | 426 Wh kg^-1^ (at 0.4 mA cm^-2^) |
